# Unmasking a Recessive Allele by a Rare Interstitial Deletion at 10q26.13q26.2: Prenatal Diagnosis of *MMP21* ‐Related Disorder and Further Refine *INSYN2A* Involvement in the Postnatal Cognitive Phenotype

**DOI:** 10.1002/mgg3.70082

**Published:** 2025-02-20

**Authors:** Jiasun Su, Shujie Zhang, Wei Li, Yuan Wei, Fei Lin, Chaofan Zhou, Xianglian Tang, Yueyun Lan, Minpan Huang, Qiang Zhang, Shang Yi, Qi Yang, Sheng Yi, Xunzhao Zhou, Zailong Qin, Peng Huang

**Affiliations:** ^1^ Genetic and Metabolic Central Laboratory of Maternal and Child Health Hospital of Guangxi Zhuang Autonomous Region Nanning China; ^2^ Birth Defects Prevention and Control Institute of Guangxi Zhuang Autonomous Region Nanning China; ^3^ Guangxi Key Laboratory of Reproductive Health and Birth Defect Prevention Nanning China

**Keywords:** 10q26 microdeletion syndrome, inhibitory synaptic factor 2A, *MMP21*, unmasking recessive allele

## Abstract

**Background:**

The 10q26 microdeletion syndrome (OMIM #609625) is a distinct genomic disorder characterized by a spectrum of clinical features including craniofacial anomalies, developmental delay (DD)/intellectual disability (ID), hypotonia, cardiovascular, and urogenital malformations. Despite the identification of critical regions within 10q26 linked to the syndrome's phenotype, the specific genes responsible for the associated facial characteristics, microcephaly, cognitive issues, and growth deficiencies remain elusive. Interstitial deletions at 10q25.3‐q26.3 are rare, and their contributions to 10q26 microdeletion syndrome remain unknown.

**Methods:**

We conducted trio‐whole‐exome sequencing (WES) on a fetus presenting with ventricular septal defect (VSD), aortic span, intrauterine growth retardation (IUGR), and microcephaly. Variant classification was assessed according to the American College of Medical Genetics and Genomics/Association for Molecular Pathology (ACMG/AMP) guidelines, and the causative gene associated with cognitive phenotype was refined by means of smallest regions of overlap (SRO).

**Results:**

A homozygous variant c.1544A>G (p.Tyr515Cys) in *MMP21* and a large deletion at 10q26.13‐q26.2 which unmasked the homozygous mutation were identified in the proband. The maternally inherited 10q26.13q26.2 deletion was classified as likely pathogenic, while the variant c.1544A>G was of uncertain significance (VUS) based on ACMG/AMP criteria. A critical interval of approximately *~*500 kb implicating the involving genes *DOCK1* and *INSYN2A* (inhibitory synaptic factor 2A) in the cognitive phenotype of 10q26 microdeletion syndrome was refined.

**Conclusion:**

This study introduces a recessive *MMP21* mutation unmasked by a rare 10q26.13q26.2 deletion via WES in a Chinese fetus with congenital heart disease (CHD), IUGR, and microcephaly. We further refine *INSYN2A* as a potential candidate gene for cognitive phenotype in 10q26.1‐q26.3 region. Our study also highlights the utility of WES for its advantage in simultaneously analyzing both single nucleotide variants (SNVs) and copy number variants (CNVs) and provide a reference for prenatal diagnosis and genetic counseling in patients with similar conditions.

## Graphical abstract

This study introduces a recessive *MMP21* mutation unmasked by a rare 10q26.13q26.2 deletion via WES in a Chinese fetus and further refines *INSYN2A* as a potential candidate gene for cognitive phenotype in 10q26.1‐q26.3 region, providing a reference for prenatal diagnosis and genetic counseling in patients with similar conditions.

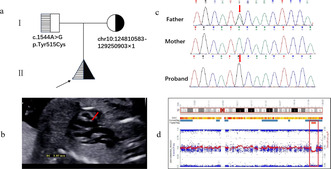

## 1 Background

The 10q26 microdeletion syndrome (OMIM #609625) is a rare and clinically heterogeneous disorder, since its initial report by Lewandowski et al. in 1963 (Lewandowski Jr et al. 1978). Several 100 cases have been described. The deletions associated with this syndrome typically ranged from 3.5 to 17 Mb in size and were often located at subterminal regions on chromosome 10 long arm (10q), and the most distal breakpoint observed was at 10q25 (Piard et al. 2014; Yatsenko et al. 2009). Common clinical features observed postnatally in affected individuals include craniofacial anomalies, developmental delay/intellectual disability (DD/ID), urinary tract abnormalities, cardiac malformations, and neurodevelopmental deficits (Courtens et al. 2006; Irving et al. 2003; Scigliano et al. 2004; Yatsenko et al. 2009). Interstitial 10q26 deletions may exhibit similar features, indicating that critical genes or regions within the deleted segment likely contribute to the syndromic manifestations. Interstitial deletions at 10q25.3‐q26.3 are rare compared to 10q26 terminal deletions. To date, only a few 10q25.3q26.3 interstitial deletion cases with available microarray data (Cherik et al. 2021; Choucair et al. 2015; Iourov et al. 2014; Miller et al. 2009; Piard et al. 2014; Ramos et al. 2016; Tosur et al. 2015; Yatsenko et al. 2009) have been reported. Due to the variability in deletion size and the nonspecific nature of clinical features, establishing a clear genotype–phenotype correlation had been challenging. Thus, identifying interstitial deletion cases involving 10q26 region may help to narrow down causal genes.

Several critical regions contributing to the specific phenotype of 10q26 deletion syndrome have been proposed, including ID, growth retardation, microcephaly, cardiac defects, and genital anomalies (Cherik et al. 2021; Chitkara et al. 2011; Choucair et al. 2015; Lin et al. 2016; Yatsenko et al. 2009). Many genes in the 10q26 region have been discussed in relation to this disorder, but the exact relationships remain unclear.

Studies suggest that copy number variants (CNVs) may contribute to clinical phenotypes through a recessive mode of gene action (Bedeschi et al. 2010; Egloff et al. 2018; Hochstenbach et al. 2012; Schwartz et al. 2018). Several hypotheses have been proposed to explain the phenotypic variability between parents and offspring carrying the same genomic imbalance, including unmasking of a recessive variant by a chromosomal deletion (Egloff et al. 2018). Currently, next‐generation sequencing technologies, particularly whole‐exome sequencing (WES), provide a unique opportunity to search for additional single nucleotide variants (SNVs) that may contribute to the variable phenotypic expression of inherited CNVs.

Herein, we present a rare maternal origin 10q26.13q26.2 interstitial deletion unmasking a *MMP21* recessive mutation in a Chinese fetus with congenital heart disease (CHD), intrauterine growth restriction (IUGR), and microcephaly. This finding could help improve the recognition of genes involved in this region and highlight the clinical utility of exome sequencing in enabling the simultaneous detection of point mutations and CNVs.

## 2 Materials and Methods

### 2.1 Ethical Compliance

This study was approved by the Medical Ethics Committee of the Maternal and Child Health Hospital of Guangxi Autonomous Region. Written informed consent was obtained from the family for publication of their pertinent images included in this paper.

### 2.2 DNA Extraction

The genomic DNA was extracted from the parent's blood and their fetus tissue using QIAamp DNA Kit (Qiagen, Germany). DNA concentration and quality were determined by Q‐bit (Thermo Fisher Scientific, Waltham, MA). Approximately 200 ng of genomic DNA from each sample was randomly fragmented into 150‐ to 200‐basepair length by ultrasonicator (M220, Covaris).

### 2.3 Whole‐Exome Sequencing (WES)

WES analysis was performed at parent's request. DNA library was constructed by Agilent SureSelect Human Exon V5 kit (Agilent Technologies, Santa Clara, CA) according to the standard manufacturer's protocol. Sequencing was processed on Illumina HiSeq X Ten System (Illumina Inc., CA) based on the manufacturer's protocols. The sequencing reads were mapped to the Genome Reference Consortium Human genome build 37 (GRCh37). The Genome Analysis Toolkit (GATK) was used for variant calling. SNVs and insertion–deletions (indels) were saved as VCF format files and uploaded to the online variation annotation tool TGex (Life Map Sciences Ltd, Israel) for further filtering and prioritizing. Common variants were filtered based on the frequencies in the Exome Sequencing Project (https://esp.gs.washington.edu), the 1000G (http://www.1000genomes.org), GnomAD (http://gnomad‐sg.org/), and local database. The variant pathogenicity was assessed according to the American College of Medical Genetics and Genomics/Association for Molecular Pathology (ACMG/AMP) guidelines (Richards et al. 2015).

### 2.4 Variant Validation

Sanger sequencing was performed for the validation of the *MMP21* variants identified by WES. The primers for the amplification of exonic regions of the *MMP21* gene (NM_14711.1) were designed by the UCSC ExonPrimer (https://ihg.helmholtz‐munich.de/cgi‐bin/primer/ExonPrimerUCSC.pl?db=hg19&acc=uc001liu.3), the specificity and reliability of primers were evaluated by online UCSC In Silico PCR. The primers designed for the candidate variation (c.1544A>G) were listed as follows: Left (5′‐3′): AAGAAGTTCAAGTCTAAAGAATTTGG, and right (5′‐3′): TCCGGTTTTCTTTGTAAACAGTATC. The primers were synthesized by Invitrogen Biotechnology, Shanghai, China. Polymerase chain reaction (PCR) analysis was performed (Takara Biotechnology) and products were sequenced by Thermo Fisher Scientific, Guangzhou, China. Sequence alignment was performed by Chromos version 2.5.0. The deletion detected by WES at 10q26 interstitial region was validated by using Illumina HumanCytoSNP‐12 v2.1 BeadChip (Illumina, USA) for chromosomal microarray analysis.

## 3 Results

### 3.1 Clinical Information and Ultrasound Findings

**FIGURE 1:**
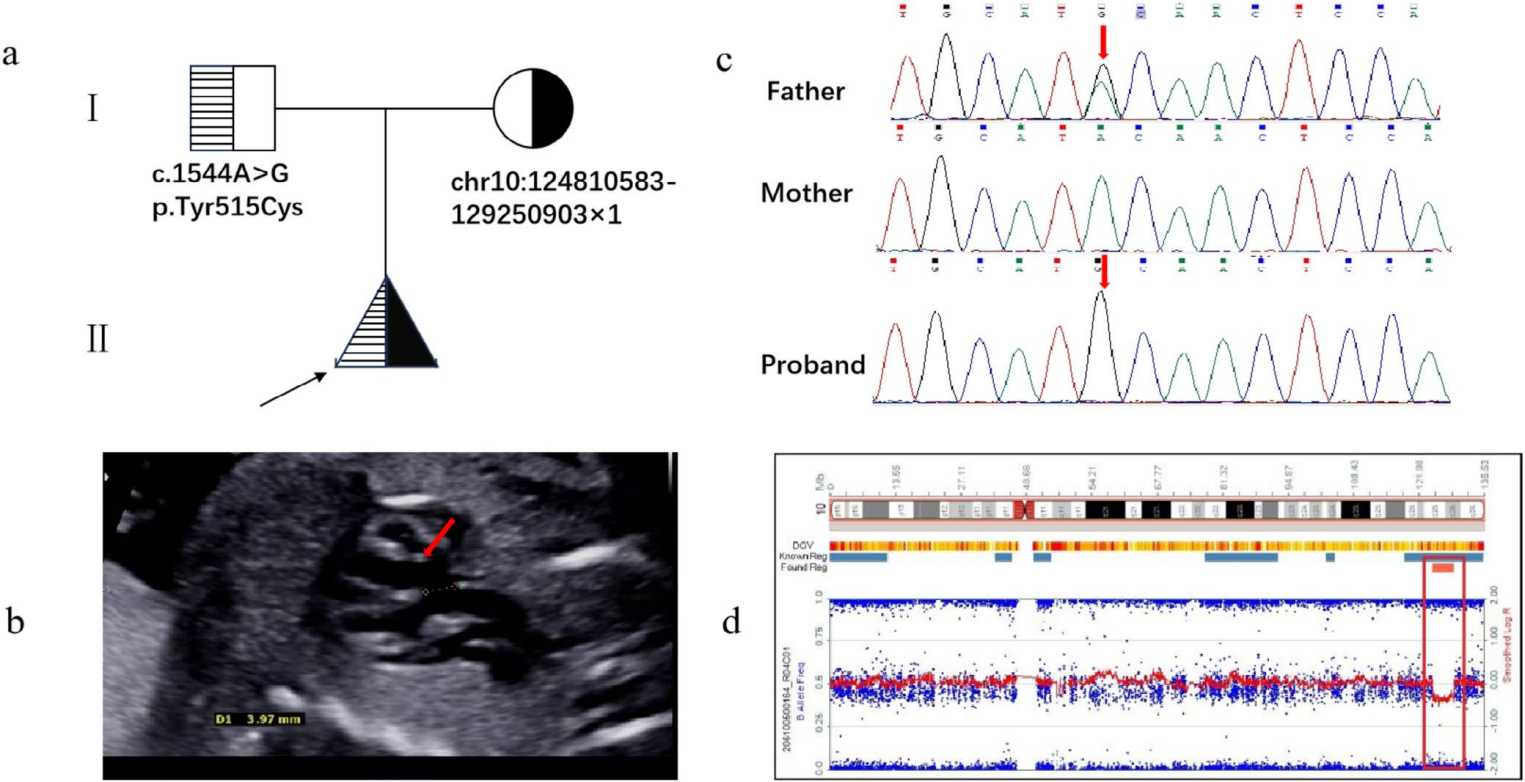
The ultrasound images and the identification of the variants involving *MMP21*. (a) Pedigree of family. The variant of *MMP21*, NM_147191.1: c.1544 A>G in exon 7 in the proband was inherited from the father (c) and confirmed by Sanger sequencing, the 10q26 deletion detected by WES confirmed by CMA (d) was inherited from the mother. (b) ultrasonographic examination indicated fetal ventricular septal defect.

A 24‐year‐old, gravida 1, para 0 woman was first referred for genetic counseling at 23 weeks of gestation due to detected fetal abnormalities on prenatal ultrasound. The woman presented with mild ID, language impairment, short stature (145 cm in height), and polydactyly of the right hand. Unfortunately, she could not provide information about her pregnancy, birth weight, birth height, and head circumference due to her cognitive conditions. Her husband was 32 years of age and healthy. Prenatal ultrasound revealed a ventricular septal defect (VSD) (Figure 1), and subsequent examinations indicated IUGR and microcephaly. Umbilical cord blood sampling was performed for this pregnancy and WES was performed at family's request. The woman opted for terminating of pregnancy (TOP) in another hospital and postnatal autopsy was unavailable.

### 3.2 Variant Analysis and Validation

A large heterozygous deletion at 10q26.13‐q26.2 (chr10:124810583–129250903) was revealed in both the fetus and the mother through trio‐WES, indicating that this deletion was maternal origin. This deletion can be classified as likely pathogenic. Simultaneously, an apparent homozygous missense variant c.1544A>G/p. Tyr515Cys in *MMP21* gene in this fetus was identified, this variant was reported as a single nucleotide polymorphism (rs370620506) in the dbSNP database and can be classified as a variant of uncertain significance according to the ACGM/AMP guidelines. The homozygous mutation is actually a hemizygous mutation unmasked by the heterozygous deletion, trio‐WES revealed that variant c.1544A>G was inherited from the father and validated by Sanger sequencing (Figure 1). No other candidate variants matched with known phenotypes for the fetus and the mother were extracted.

## 4 Discussion

The *MMP21* gene (OMIM #608416) encodes a member of the matrix metalloproteinase family and plays a crucial role in various physiological and pathological processes (Oh et al. 2017; Zhao et al. 2014). Mutations in *MMP21* are linked to early embryonic development disorders, including HTX7 (autosomal visceral heterotaxy‐7, OMIM #616749), HTX (Heterotaxy syndrome), and CHD (Guimier et al. 2015). Previous studies have reported homozygous or compound heterozygous *MMP21* mutants in patients with HTX (Guimier et al. 2015; Li et al. 2019; Liu et al. 2020; Yuan et al. 2020), indicating that biallelically damaging variants of *MMP21* are significant in the left–right patterning of HTX. Zhuang‐Zhuang Yuan et al. reported a nonsense *MMP21* variant (c.G496T, p.G166*) causing Dextrocardia and CHD in a Han Chinese Patient (Yuan et al. 2020), Xi‐ji Qin et al. identified three *MMP21* heterozygous missense variants, c.731G>A (p.G244E), c.829C>T (p.L277F), and c.1459A>G (p.K487E) in three unrelated Chinese Han patients with HTX and complex CHD. Functional analysis indicated that the underlying mechanism of HTX and CHD may not be the haploinsufficiency of *MMP21*, although loss of function in *MMP21* can cause the phenotypes of situs inversus with CHD in mice with chemically induced missense mutations c.677T>C, p.(Ile226Thr) or p.(Ala321Pro) (Qin et al. 2022). The clinical heterogeneity of *MMP2*1‐related diseases was evident, with cardiovascular manifestations indicating the multifunctional properties of *MMP21*. The fetus we presented here exhibited VSD, not meeting the core phenotype of HTX. Furthermore, the variant (c.1544A>G/p. Tyr515Cys) in *MMP21* was a VUS, at the time of the interpretation, there was not sufficient evidence to determine whether the variant was related to disease or not. However, attributed to the presence of a large deletion occurring on the homologous chromosome, the recessive variant became unmasked and the involvement in this patient's phenotype might not be excluded regarding the high heterogeneity of *MMP21*‐related disorders.

Interstitial deletions at 10q25.3‐q26.3 are rare. To date, only a dozen cases of 10q25.3‐q26.3 interstitial deletion with available microarray data have been documented in the literature from our observation (Cherik et al. 2021; Choucair et al. 2015; Iourov et al. 2014; Miller et al. 2009; Piard et al. 2014; Ramos et al. 2016; Tosur et al. 2015; Yatsenko et al. 2009). We listed the clinical feature of 10q25.3‐q26.3 interstitial deletion cases in Table 1. Neurologic anomalies are the most consistent feature observed across all reported patients, followed by other common features including craniofacial dysmorphism, genital anomalies, hypotonia, hearing loss, CNS malformations, urinary tract/renal anomalies, growth retardation, cardiovascular anomalies, and limb anomalies. The collective data support the notion that more than one critical region is responsible for the variable clinical presentation of 10q26 deletion syndrome. Additionally, VSD was relatively low in frequency (0/10) in 10q25.3‐q26.3 interstitial deletion patients, this might be attributed to the small number of cases reported and a lack of sufficient details, more cases would be needed to quantify the prevalence. Our findings provide a rare reference for prenatal diagnosis and genetic counseling for families identified in the future.

**TABLE 1:** Clinical features of the 12 patients with 10q25.3‐q26.3 interstitial deletions reported previously and this study.

Clinical findings	Yatsenko et al. (2009)				Miller et al. (2009)	Piard et al. (2014)	Iourov et al. (2014)	Choucair et al. (2015)	Ramos et al. (2016)	Tosur et al. (2015)	Cherik et al. (2021)		Total	Present study	
	Case 1	Case 3	Case 4	Case 5	Case 4						Case 1	Case 2	12	Proband	Mother
Gender	Male	Male	Female	Male	Male	Male	Male	Male	Male	Male	Male	Female		Male	Female
Age/GA	29 ws	3 ys	2 ys	25 ys	32 ms	32 ms	2.3 ys	6 ys	9 ys	17 ms	21.5 ms	23 ys		31 ws	24 ys
Deletion region	10q26.13q26.3	10q26.2q26.3	10q26.12q26.2	10q26.12q26.2	q25.3q26.13	10q25.3q26.12	10q26.2q26.3	10q26.1	10q26.11q26.13	10q25.3q26.13	10q26.2	10q26.2		10q26.13q26.2	10q26.13q26.2
Deletion interval^a^	Chr10:116263488–133658347	Chr10:128256930–131946100	Chr10:122888789–128830242	Chr10:122888789–128830242	Chr10:118506755–125492105	Chr10:120656863–124511193	Chr10:128190760–133998503	Chr10:119512117–124082152	Chr10:120073905–125602943	Chr10:117012878–125217066	Chr10:128771757–129634868	Chr10:128771757–129634868		Chr10:124811728–129272635	Chr10:124811728–129272635
Size (Mb)	17.22	3.51	5.8	5.8	6.99	3.85	5.8	4.5	5.5	8.2	0.86	0.86		4.46	4.46
Inheritance	Na	Na	Paternal	Na	De novo	De novo	Na	De novo	De novo	De novo	Maternal	Na		Maternal	Na
Growth	+	−	+	−	+	−	−	−	−	−	+	+	5/12	Na	Na
IUGR	+	Na	Na	Na	Na	−	−	−	−	−	+	−	2/8	+	Na
Short stature	Na	−	+	−	+	−	−	−	−	−	+	+	4/11	Na	+
Craniofacial dysmorphism	+	+	+	+	+	+	−	+	−	+	+	+	10/12		
Microcephaly	+	−	+	+	+	+	−	+	−	+	+	+	9/12	+	Na
Broad nasal bridge/Prominent nose	+	+	+	+	−	−	−	+	Na	+	+	+	8/11	Na	Na
Ear anomalies	+	+	+	+	+	−	−	+	−	−	+	+	8/12	Na	Na
Hypertelorism	+	−	+	+	−	−	−	−	−	+	−	−	4/12	Na	Na
Eye anomalies	−	+	−	−	+	−	−	−	−	+	+	+	5/12	Na	Na
Neurologic anomalies	+	+	+	+	+	+	+	+	+	+	+	+	12/12		
ID	+	NA	+	+	M	+	NA	−	+	NA	NA	+	6/7	NA	+
DD	+	+	+	+	+	+	M	+	+	+	+	+	12/12	Na	+
Behavioral disorders	+	−	+	+	Na	Na	Na	−	+	Na	−	−	4/8	Na	−
Limb anomalies	−	+	+	Na	+	Na	−	+	+	−	+	+		Na	+
Brachydactyly	−	−	−	Na	−	Na	−	−	−	−	−	−			+
Clinodactyly	−	+	+	Na	+	Na	−	−	+	−	+	−			
Feet	Na	Na	Na	Na	Na	Na	Na	Flat feet	Na	Na	Club feet	Hollow feet	3/3		−
Cardiovascular anomalies	**+**	−	NA	+	−	−	NA	−	ELV	+	−	−	4/10	+	
PDA	+	−	Na	+			Na			+			3/10		
VSD	−	−	Na				Na						0/10	+	−
ASD	−	−	Na				Na			+			1/10		
Other anomalies							−								
Urinary tract/renal anomalies	+	Na	Na	Na	Na	+	−	Na	Na	Na	+	−	3/5	Na	Na
Genital anomalies	+	Na	Na	Na	+	+	−	+	+	+	−	−	6/9	Na	Na
Hearing loss	+	Na	Na	Na	+	Na	−	−	+	+	Na	Na	4/6	Na	−
CNS malformations	+	−	Na	Na	+	−	−	−	Mild	Na	−	Na	3/7	Na	Na
Hypotonia	Na	+	Na	Na	+	Na	−	+	+	+	+	Na	6/7	Na	−

Abbreviations: −, feature absent; +, feature present; ASD, atrial septal defect; CNS, central nervous system; DD, developmental delay; ELV, enlarged left ventricle; GA, gestational age; ID, intellectual disability; IUGR, intrauterine growth retardation; M, moderate; m, month; na, not available; PDA, patent ductus arteriosus; VSD, ventricular septal defect; w, week; y, year.

a Deletion intervals were shown according to the human Feb. 2009 (GRCh37/hg19) assembly.

**FIGURE 2:**
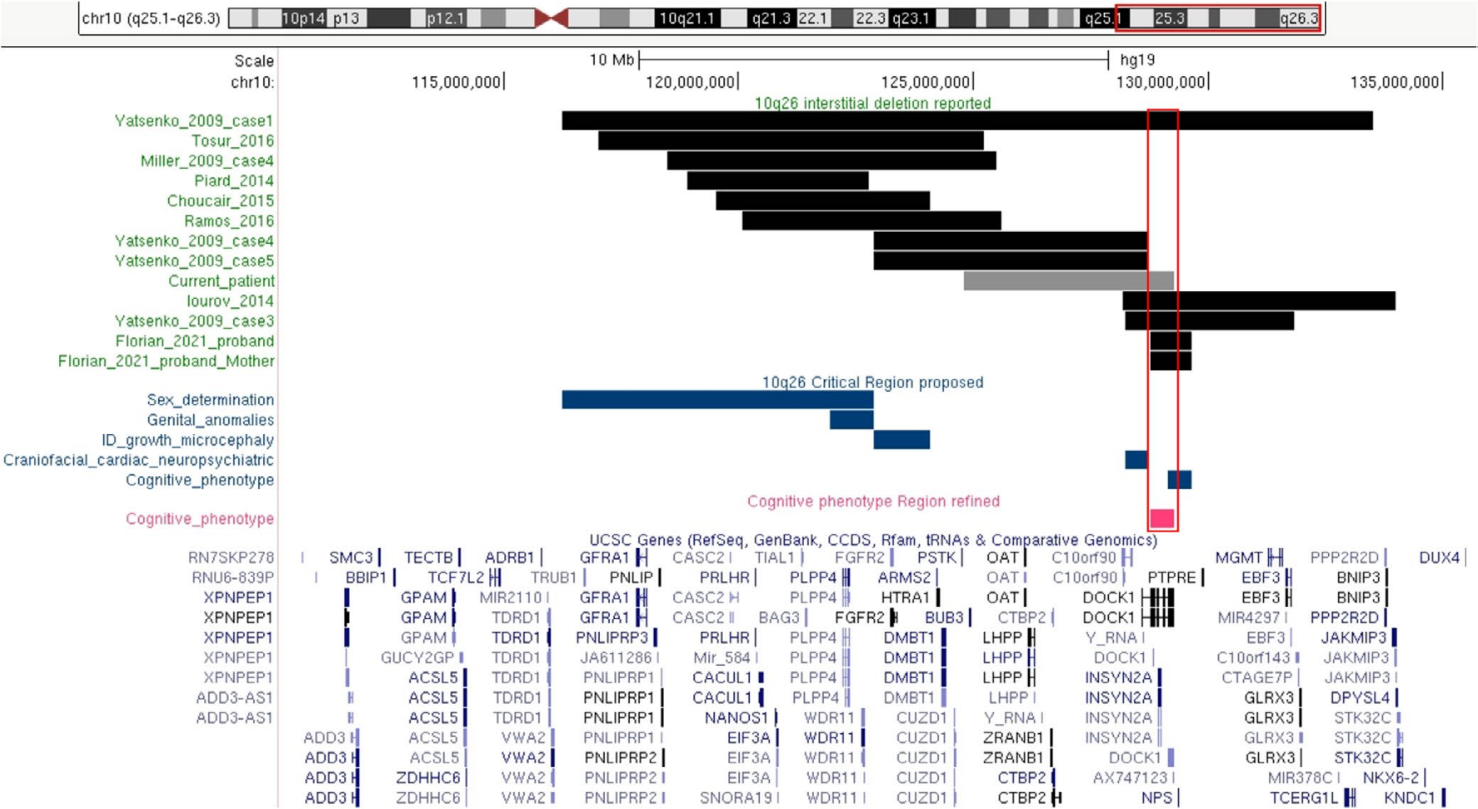
The schematic presentation referred to interstitial deletion at 10q26 region. Black bars: Cases with detailed size and location with known coordinates of the deletion in the literature and our cases. Blue bars: Critical regions proposed for specific phenotypic features of 10q26 deletion syndrome. Red bars: The cognitive phenotype region refined by smallest regions overlapped (UCSC Genome Browser, hg19).

Several critical regions have been proposed to contribute to the specific phenotype of 10q26 deletion syndrome (Figure 2, blue bars). Choucair et al. proposed a critical region between 122878779 and 124082152 for the facial characteristics, ID, growth retardation, and microcephaly. This region contains five protein‐coding genes (*ATE1*, *BTBD16*, *FGFR2*, *NSMCE4A*, *TACC2*), but it remained unclear which gene or combination of genes is responsible for these features. They further narrowed down the critical to a *~*930‐kb interval at 10q26.12 between 121946873 and 122878779, which encompasses two genes, *PPAPDC1A* and *WDR11*, implicated in genital abnormalities (Choucair et al. 2015). Yatsenko et al. proposed a *~*600‐kb region involving the *C10orf90* and *DOCK1* genes associated with craniofacial dysmorphism, CHD and DD/ID. The *DOCK1* gene was considered as a candidate gene in the pathogenesis of cardiovascular phenotype (Yatsenko et al. 2009). Florian et al. suggested that haploinsufficiency of *DOCK1* was the primary cause of 10q26.2 microdeletion syndrome (Cherik et al. 2021).

The proband's mother harbored a deletion overlapping with the interstitial deletion in Cherik's patients, which included the *DOCK1* gene, the smallest overlapping region between these cases excludes the involvement of *NPS* gene (OMIM # 609513) in the cognitive phenotype (Figure 2, red bar). This allows for the refinement of a *~*500‐kb critical interval for the cognitive phenotype of 10q26 microdeletion syndrome, which includes only two coding genes, *DOCK1* and *INSYN2A* (inhibitory synaptic factor 2A). Although the *DOCK1* gene has been proposed as the major candidate gene in the 10q26 deletion syndrome (Cherik et al. 2021), we suppose its pLI (probability of loss‐of‐function intolerance) score (0) suggests that it is unlikely to cause a dominant disease. *INSYN2A* (inhibitory synaptic factor 2A, OMIM #617129) encodes a postsynaptic density protein involved in synaptic inhibition, it remains a protein of unknown function, recently research has demonstrated that this protein regulates postsynaptic inhibition in the newborn mouse brain and could contribute to developmental brain disorders (Uezu et al. 2016), it is predicted to be a haploinsufficient gene with the pLI score of 0.91 (DECIPHER). Neither point mutations nor chromosome aberrations exclusively affecting *INSYN2A* have been reported to date. Indeed, the evidence above is anecdotal, more cases and further functional research would be needed to prove this hypothesis.

IUGR and microcephaly are among the conditions observed in individuals with 10q26 interstitial deletions (Table 1). It is possible that a fetus with this deletion may exhibit a mix of phenotype associated with disorders linked to the *MMP21* gene, in addition to the characteristics of the 10q26 deletion syndrome itself. The deletion's effect on the fetus could potentially unmask a variant of the *MMP21* gene, leading to cardiovascular anomalies. This is suggested by the fact that the mother, who also carries the deletion, did not display any cardiovascular issues during her cardiac ultrasound examination. However, it remains uncertain whether the cardiovascular phenotype in proband's mother shows reduced penetrance, given that such anomalies are not uncommon among patients with 10q25.3q26.3 interstitial deletions (Table 1).

Additionally, the specific deletion in question, spanning chr10:124810583–129250903, was initially deemed to be of uncertain significance according to the ClinGen CNV Interpretation Scoring Rubric (https://cnvcalc.clinicalgenome.org/cnvcalc/cnv‐loss). This classification was made without taking into account our cases. However, by incorporating previously documented cases, such as the DECIPHER patient with the identifier 288544, along with the protein‐coding RefSeq genes present in the region, the cumulative score surpassing the threshold deletion could be revised to a likely pathogenic effect.

In conclusion, we present a rare case of a heterozygous interstitial deletion at 10q26.13‐q26.2 that revealed a recessive variant of the *MMP21* gene. The ability to detect both large and small SNV changes within 10q26 region underscores the value of exome sequencing in clinical diagnostics. Our study advances the understanding of the 10q26 deletion syndrome and disorders related to the *MMP21* gene. We further narrow down the smallest critical region linked to cognitive phenotypes and reinforce the hypothesis that *INSYN2A* is a potential candidate gene for DD/ID within this region. Further cases are needed to confirm the genotype–phenotype correlation, and functional analyses will be essential to better understand the underlying mechanism.

## Author Contributions

Jiasun Su and Peng Huang wrote the manuscript. Jiasun Su and Zailong Qin conceived and designed the experiments. Wei Li, Yuan Wei, Chaofan Zhou, Xianglian Tang, and Yueyun Lan performed the microarray analysis. Shujie Zhang, Qiang Zhang, Qi Yang, Sheng Yi, Minpan Huang, and Xunzhao Zhou performed the WES experiments. Fei Lin and Peng Huang performed the Sanger sequencing experiments. Shang Yi and Jiasun Su analyzed the data. Zailong Qin and Peng Huang helped to revise the manuscript.

## Conflicts of Interest

The authors declare no conflicts of interest.

## Data Availability

The data that support the findings of this study are available on request from the corresponding author. The data are not publicly available due to privacy or ethical restrictions.
